# Supplementary material for: Trade-off and flexibility in the dynamic regulation of the cullin-RING ubiquitin ligase repertoire
Source: PLoS Comput Biol. 2017 Nov 17;13(11):e1005869. doi: 10.1371/journal.pcbi.1005869 (PMC5711038; doi:10.1371/journal.pcbi.1005869)
Supplement: S1 Text — In S1 Text we conduct a steady state / time scale analysis of Eq (3) and provide the derivations of Eqs (5)–(9). (PDF) [file pcbi.1005869.s001.pdf]

Supporting Information: S1 Text

Steady state and time scale analysis of the Cand1 cycle model

Ronny Straube, Meera Shah, Dietrich Flockerzi, Dieter A. Wolf

Contents

S1 Steady state analysis **S2**
S1.1 Variable transformation . . . S2
S1.2 Steady state equations . . . S3
S1.2.1 Asymptotic analysis . . . S3
S1.2.2 Derivation of Eq. (S13) . . . S6
S1.2.3 Derivation of Eq. (S21) . . . S7
S1.2.4 Derivation of Eq. (S23) . . . S7
S1.2.5 Derivation of Eq. (S24) . . . S9

S2 Time scale analysis **S10**
S2.1 Eigenvalues of S . . . S10
S2.1.1 Asymptotic analysis for $\mathrm{Cand1_T} \gg \mathrm{Cul1_T}$ . . . S11
S2.1.2 Asymptotic analysis for $\mathrm{Cand1_T} \ll \mathrm{Cul1_T}$ . . . S12
S2.2 Eigenvalues of U . . . S13
S2.3 Derivation of Eqs. (S77) and (S78) . . . S16

S1 Steady state analysis

S1.1 Variable transformation

To derive explicit expressions for the steady state curves and to conduct a subsequent time-scale analysis of the ODE system in Eq. (3) it will be helpful to introduce more suitable variables. To this end, we note that since the two SRs are assumed to bind with the same affinity to Cul1 the concentrations of the binary and ternary complexes are proportional at steady state, i.e.

$$y_2 = \lambda y_1 \quad \text{and} \quad y_5 = \lambda y_4, \quad \lambda = \frac{\mathrm{SR2_T}}{\mathrm{SR1_T}}\,. \tag{S1}$$

This motivates to use the linear combinations

$$s_1 = y_2 - \lambda y_1 \quad \text{and} \quad s_2 = y_5 - \lambda y_4 \tag{S2}$$

as new variables. In addition we set

$$x = y_1 + y_4 \tag{S3}$$

$$\sigma = y_1 + y_2 + y_3 + y_4 + y_5 \tag{S4}$$

so that the inverse relations are given by

$$\begin{aligned} y_2 &= s_1 + \lambda y_1 \\ y_3 &= \sigma - [s_1 + s_2 + (1+\lambda)\,x] \\ y_4 &= x - y_1 \\ y_5 &= s_2 + \lambda\,(x - y_1)\,. \end{aligned} \tag{S5}$$

Substituting Eqs. (S2) - (S5) into Eqs. (3) yields the ODE system in new coordinates

$$\begin{aligned} \frac{ds_1}{dt} &= -k_{sr}\left(\frac{[Cul1]\,(s_1+s_2)}{K_{sr}} + s_1\right) - \beta k'_{sr}\left(\frac{[Cand1]s_1}{\eta K'_{sr}} - s_2\right) \\ \frac{ds_2}{dt} &= \beta k'_{sr}\left(\frac{[Cand1]s_1}{\eta K'_{sr}} - s_2\right) - k'_{sr}\left(\frac{(s_1+s_2)\,(\sigma - [s_1+s_2+(1+\lambda)\,x])}{K'_{sr}} + s_2\right) \\ \frac{dx}{dt} &= k_{sr}\left(\frac{[Cul1][SR1]}{K_{sr}} - y_1\right) + k'_{sr}\left(\frac{[SR1]\,(\sigma - [s_1+s_2+(1+\lambda)\,x])}{K'_{sr}} - (x - y_1)\right) \\ \frac{dy_1}{dt} &= k_{sr}\left(\frac{[Cul1][SR1]}{K_{sr}} - y_1\right) - \beta k'_{sr}\left(\frac{[Cand1]y_1}{\eta K'_{sr}} - (x - y_1)\right) \\ \frac{d\sigma}{dt} &= k_{sr}\left(\frac{[Cul1]\,[(1+\lambda)\,[SR1] - (s_1+s_2)]}{K_{sr}} - [s_1 + (1+\lambda)\,y_1]\right) \\ &\quad + \alpha k_{sr}\left(\frac{[Cul1][Cand1]}{\eta K_{sr}} - (\sigma - [s_1+s_2+(1+\lambda)\,x])\right) \end{aligned} \tag{S6}$$

where the mass conservation relations (Eq. 4) are now given by

$$\begin{aligned} [Cul1] &= \mathrm{Cul1_T} - \sigma \\ [SR1] &= \mathrm{SR1_T} - x \\ [SR2] &= \mathrm{SR2_T} - (s_1 + s_2 + \lambda x) \\ [Cand1] &= \mathrm{Cand1_T} - \sigma + (1+\lambda)\, y_1 + s_1 . \end{aligned} \tag{S7}$$

S1.2 Steady state equations

By construction, we have

$$s_1 = s_2 = 0 \tag{S8}$$

at steady state (cf. Eqs. S1 and S2). In addition, we know that (due to the validity of detailed balance) each term in brackets in Eqs. (2) has to vanish separately. Specifically, we shall use the following relations

$$\begin{aligned} [Cul1]\,[Cand1] &= \eta K_{sr} y_3 \\ y_1\,[Cand1] &= \eta K'_{sr} y_4 \\ [Cul1]\,[SR1] &= K_{sr} y_1 . \end{aligned}$$

With the help of Eqs. (S5), (S7) and (S8) these relations can be written in the form

$$(\mathrm{Cul1_T} - \sigma)\,(\mathrm{Cand1_T} - \sigma + (1+\lambda)\, y_1) = \eta K_{sr}\,(\sigma - (1+\lambda)x) \tag{S9}$$

$$y_1\,(\mathrm{Cand1_T} - \sigma + (1+\lambda)\, y_1) = \eta K'_{sr}\,(x - y_1) \tag{S10}$$

$$(\mathrm{Cul1_T} - \sigma)\,(\mathrm{SR1_T} - x) = K_{sr} y_1 . \tag{S11}$$

Together, Eqs. (S9) - (S11) constitute 3 coupled nonlinear equations for x, y_1 and σ.

S1.2.1 Asymptotic analysis

In the following we derive approximate expressions for x, y_1 and σ that are valid in the limit $\eta K_{sr} \ll \mathrm{Cul1_T}$ (cf. Table 1). In addition, we shall be mostly interested in the physiologically relevant case $\mathrm{SR_T} > \mathrm{Cul1_T}$ [1] where Cul1 is saturated with substrate receptors.

We begin by rewriting Eq. (S9) which yields the quadratic equation

$$\begin{aligned} &\sigma^2 - \sigma\,(\mathrm{Cul1_T} + \mathrm{Cand1_T} + (1+\lambda)\, y_1 + \eta K_{sr}) \\ &+ \mathrm{Cul1_T}\,(\mathrm{Cand1_T} + (1+\lambda)\, y_1) + \eta K_{sr}\,(1+\lambda)\, x = 0 . \end{aligned} \tag{S12}$$

In the limit $\eta K_{sr} \ll \mathrm{Cul1_T}$ its solution can be approximated by (see below for a derivation)

$$\sigma \approx \begin{cases} \mathrm{Cul1_T} - \eta K_{sr} \frac{\mathrm{Cul1_T} - (1+\lambda)x}{\mathrm{Cand1_T} - \mathrm{Cul1_T} + (1+\lambda)y_1} & \mathrm{Cand1_T} > \mathrm{Cul1_T} - (1+\lambda)\, y_1 \\ \mathrm{Cand1_T} + (1+\lambda)\, y_1 - \eta K_{sr} \frac{\mathrm{Cand1_T} + (1+\lambda)(y_1 - x)}{\mathrm{Cul1_T} - (\mathrm{Cand1_T} + (1+\lambda) y_1)} & \mathrm{Cand1_T} < \mathrm{Cul1_T} - (1+\lambda)\, y_1 \end{cases} . \tag{S13}$$

Substituting the expressions from Eq. (S13) into Eq. (S10) yields

$$x \approx \begin{cases} y_1 \left(1 + \frac{\mathrm{Cand1_T} - \mathrm{Cul1_T} + (1+\lambda) y_1}{\eta K'_{sr}}\right) & \mathrm{Cand1_T} > \mathrm{Cul1_T} - (1+\lambda)\, y_1 \\ y_1 \left(1 + \frac{K_{sr}}{K'_{sr}} \frac{\mathrm{Cand1_T} + (1+\lambda)(y_1 - x)}{\mathrm{Cul1_T} - (\mathrm{Cand1_T} + (1+\lambda) y_1)}\right) & \mathrm{Cand1_T} < \mathrm{Cul1_T} - (1+\lambda)\, y_1 \end{cases} . \tag{S14}$$

The case $\mathbf{Cand1_T} < \mathbf{Cul1_T} - (1+\lambda)y_1$

When $\mathrm{Cand1_T} < \mathrm{Cul1_T} - (1+\lambda)y_1$ the terms $\sim \mathcal{O}(\eta K_{sr})$ and $\sim \mathcal{O}(K_{sr}/K'_{sr}) \sim \mathcal{O}(10^{-6})$ can be neglected so that substituting the respective expressions from Eqs. (S13) and (S14) into Eq. (S11) yields a quadratic equation for y_1 given by:

$$y_1^2 - y_1 \left(\mathrm{SR1_T} + \frac{\mathrm{Cul1_T} - \mathrm{Cand1_T} + K_{sr}}{1+\lambda}\right) + \mathrm{SR1_T} \frac{\mathrm{Cul1_T} - \mathrm{Cand1_T}}{1+\lambda} = 0. \tag{S15}$$

In the limit $K_{sr} \ll \mathrm{Cul1_T}$ the solution of this equation can be approximated [2]

$$y_1 \approx \begin{cases} \mathrm{SR1_T}\,, & \mathrm{SR1_T} < \mathrm{Cul1_T} - (\mathrm{Cand1_T} + \mathrm{SR2_T}) \\ \frac{\mathrm{SR1_T}}{\mathrm{SR1_T} + \mathrm{SR2_T}} (\mathrm{Cul1_T} - \mathrm{Cand1_T})\,, & \mathrm{SR1_T} > \mathrm{Cul1_T} - (\mathrm{Cand1_T} + \mathrm{SR2_T}) \end{cases} . \tag{S16}$$

Hence, as long as Cul1 is not saturated (by SRs and/or Cand1) the concentration of Cul1.SR1 (and similarly for Cul1.SR2) increases linearly with the total concentration of SR1 (and SR2). Beyond the saturation point the concentration of the SCF complexes only increases proportional to the relative SR abundances. In addition, the total cullin concentration is reduced by Cand1 which sequesters part of Cul1 into Cul1.Cand1 complexes.

The case $\mathbf{Cand1_T} > \mathbf{Cul1_T} - (1+\lambda)y_1$

In this case we have to substitute the full expressions from Eqs. (S13) and (S14) into Eq. (S11) which yields a 4th order polynomial equation in the rescaled variable

$$y = (1+\lambda)\frac{y_1}{\mathrm{Cul1_T}}, \quad \lambda = \frac{\mathrm{SR2_T}}{\mathrm{SR1_T}} \tag{S17}$$

that can be written in the form

$$y^4 + 2y^3 \left(\frac{\eta K'_{sr} + \mathrm{Cand1_T} - \mathrm{Cul1_T}}{\mathrm{Cul1_T}}\right) + a_2 y^2 + a_1 y + \left(\frac{\eta K'_{sr}}{\mathrm{Cul1_T}}\right)^2 \frac{\mathrm{SR_T}}{\mathrm{Cul1_T}} = 0 \tag{S18}$$

where $\mathrm{SR_T} = \mathrm{SR1_T} + \mathrm{SR2_T} = \mathrm{SR1_T}(1+\lambda)$ denontes the total SR concentration and the coefficients a_2 and a_1 are given by

$$\begin{aligned} a_2 &= -\left[\frac{\eta K'_{sr}}{\mathrm{Cul1_T}}\left(\frac{K'_{sr} + \mathrm{Cul1_T} + \mathrm{SR_T}}{\mathrm{Cul1_T}}\right) - \left(\frac{\eta K'_{sr} + \mathrm{Cand1_T} - \mathrm{Cul1_T}}{\mathrm{Cul1_T}}\right)^2\right] \\ a_1 &= -\left(\frac{\eta K'_{sr}}{\mathrm{Cul1_T}}\right)\left[\frac{\mathrm{Cand1_T} - \mathrm{Cul1_T}}{\mathrm{Cul1_T}}\frac{\mathrm{Cul1_T} + \mathrm{SR_T} + K'_{sr}}{\mathrm{Cul1_T}} + \frac{\eta K'_{sr}}{\mathrm{Cul1_T}}\left(1 + \frac{\mathrm{SR_T}}{\mathrm{Cul1_T}}\right)\right]. \end{aligned} \tag{S19}$$

Eq. (S18) determines the steady state concentration of Cul1.SR1 under conditions when Cul1 is saturated by SRs and/or Cand1 (i.e. $\mathrm{SR_T} + \mathrm{Cand1_T} > \mathrm{Cul1_T}$).

In the following we are specifically interested in approximate solutions for the cases

$$(i)\ \mathrm{Cand1_T} \ll \mathrm{Cul1_T} \quad \text{and} \quad (ii)\ \mathrm{Cand1_T} \gg \mathrm{Cul1_T}\,. \tag{S20}$$

In the first case the solution of Eq. (S18) can be approximated by (see below for a derivation)

$$y_s \approx 1 - \frac{\mathrm{Cand1_T}}{\mathrm{Cul1_T}}\frac{K'_{sr} + \mathrm{SR_T} - \mathrm{Cul1_T}}{K'_{sr} + \left(1 + \frac{\eta K'_{sr}}{\mathrm{Cul1_T}}\right)(\mathrm{SR_T} - \mathrm{Cul1_T})}\,, \quad \mathrm{SR_T} > \mathrm{Cul1_T}\,. \tag{S21}$$

In the limit $\eta \ll 1$ (with $K'_{sr}/\mathrm{Cul1_T}$ fixed) this reduces to

$$y_{s\eta} \approx 1 - \frac{\mathrm{Cand1_T}}{\mathrm{Cul1_T}}\,. \tag{S22}$$

After rescaling according to Eq. (S17) this expression agrees with the second line in Eq. (S16). In the opposite limit ($\mathrm{Cand1_T} \gg \mathrm{Cul1_T}$) the approximate solution reads (see below for a derivation)

$$y_l \approx \frac{\eta K'_{sr}}{\mathrm{Cand1_T}}\frac{\mathrm{SR_T}}{\mathrm{Cul1_T} + \mathrm{SR_T} + K'_{sr}}\,. \tag{S23}$$

Alternatively, one may analyze Eq. (S18) in the limit $\eta \ll 1$ which yields for $\mathrm{Cand1_T} > \mathrm{Cul1_T}$ the approximate solution

$$y_{l\eta} \approx \frac{\eta K'_{sr}}{\mathrm{Cand1_T} - \mathrm{Cul1_T}}\frac{\mathrm{SR_T}}{\mathrm{Cul1_T} + \mathrm{SR_T} + K'_{sr}}\,. \tag{S24}$$

In Fig. A the different approximations for $\mathrm{Cand1_T} \gg \mathrm{Cul1_T}$ (y_l and $y_{l\eta}$) and $\mathrm{Cand1_T} \ll \mathrm{Cul1_T}$ (y_s and $y_{s\eta}$) are compared with numerical solutions (black lines) for increasing values of the preferential binding parameter η. In general, the agreement is very good except near the transition point $\mathrm{Cand1_T} = \mathrm{Cul1_T}$. Note that for $\eta \ll 1$ the small η-approximation $y_{l\eta}$ provides a better approximation to the numerical solution for $\mathrm{Cand1_T} \gg \mathrm{Cul1_T}$.

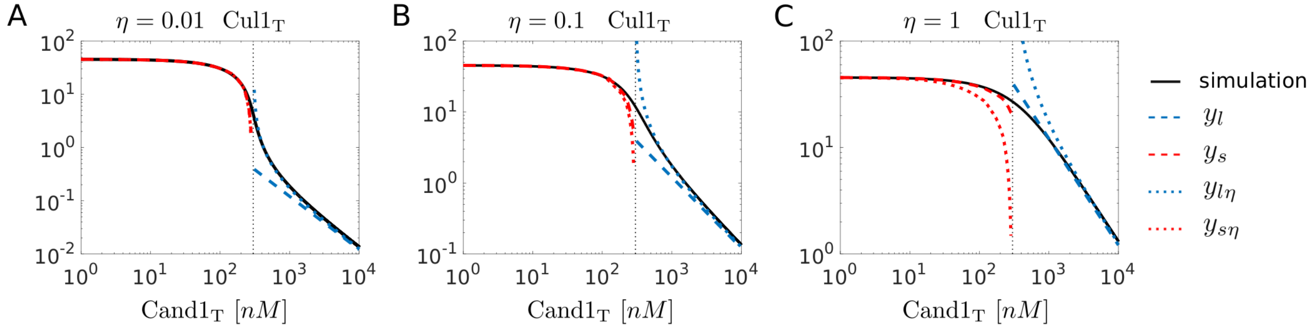

Fig A: Approximate solutions for $y_1 = y \cdot \mathrm{Cul1_T}/(1+\lambda)$ as a function of $\mathrm{Cand1_T}$. The numerical solution (black solid line) is compared with different approximations for y according to Eqs. (S21 - (S24). Vertical dotted line indicates the concentration of Cul1. Other parameters are listed in Table 1.

S1.2.2 Derivation of Eq. (S13)

Using that $\eta K_{sr} \ll \mathrm{Cul1_T}$ (cf. Table 1) we expand the solution of Eq. (S12) in the form

$$\sigma = \sigma_0 + \eta K_{sr}\sigma_1 + \mathcal{O}(\eta^2 K_{sr}^2)\,. \tag{S25}$$

Substituting this expansion into Eq. (S12) yields the set of equations

$$\sigma_0^2 - \sigma_0\left(\mathrm{Cul1_T} + \mathrm{Cand1_T} + (1+\lambda)y_1\right) + \mathrm{Cul1_T}\left(\mathrm{Cand1_T} + (1+\lambda)y_1\right) = 0 \tag{S26}$$

$$2\sigma_0\sigma_1 - \sigma_0 - \sigma_1\left(\mathrm{Cul1_T} + \mathrm{Cand1_T} + (1+\lambda)y_1\right) + (1+\lambda)x = 0\,. \tag{S27}$$

The solutions of Eq. (S26) are given by

$$\sigma_0 = \frac{\mathrm{Cul1_T} + \mathrm{Cand1_T} + (1+\lambda)\,y_1}{2} \pm \frac{\left|\mathrm{Cul1_T} - \left(\mathrm{Cand1_T} + (1+\lambda)\,y_1\right)\right|}{2},$$

i.e. there are two regimes defined by

$$I: \quad \mathrm{Cul1_T} < \mathrm{Cand1_T} + (1+\lambda)\,y_1$$

$$II: \quad \mathrm{Cul1_T} > \mathrm{Cand1_T} + (1+\lambda)\,y_1$$

and in each of these regimes there are two solutions given by

$$\sigma_0^{(1)} = \mathrm{Cul1_T} \quad \text{and} \quad \sigma_0^{(2)} = \mathrm{Cand1_T} + (1+\lambda)y_1. \tag{S28}$$

Substituting these solutions into Eq. (S27) and solving for σ_1 yields

$$\sigma_1^{(1)} = -\frac{\mathrm{Cul1_T} - (1+\lambda)\,x}{\mathrm{Cand1_T} + (1+\lambda)\,y_1 - \mathrm{Cul1_T}} \tag{S29}$$

$$\sigma_1^{(2)} = -\frac{\mathrm{Cand1_T} + (1+\lambda)\,y_1 - (1+\lambda)\,x}{\mathrm{Cul1_T} - \left(\mathrm{Cand1_T} + (1+\lambda)\,y_1\right)}.$$

To combine the solutions in Eqs. (S28) and (S29) we note that the steady state relations (S7) imply that

$$\sigma \leq \min(\mathrm{Cul1_T}, \mathrm{Cand1_T} + (1+\lambda)y_1)\,. \tag{S30}$$

To satisfy this constraint the first order corrections in Eq. (S29) must be negative. Since the numerators in Eqs. (S29) are positive we see that $\sigma_1^{(1)}$ is restricted to region I whereas $\sigma_1^{(2)}$ is restricted to region II. Hence, the solution of Eq. (S12) can be approximated by

$$\sigma \approx \begin{cases} \mathrm{Cul1_T} + \eta K_{sr}\sigma_1^{(1)} & \mathrm{Cul1_T} < \mathrm{Cand1_T} + (1+\lambda)\,y_1 \\ \mathrm{Cand1_T} + (1+\lambda)\,y_1 + \eta K_{sr}\sigma_1^{(2)} & \mathrm{Cul1_T} > \mathrm{Cand1_T} + (1+\lambda)\,y_1 \end{cases} \tag{S31}$$

which agrees with the approximation in Eq. (S13).

S1.2.3 Derivation of Eq. (S21)

To study the behavior of the solution of Eq. (S18) when $\mathrm{Cand1_T} \ll \mathrm{Cul1_T}$ we set

$$\rho = \frac{\mathrm{Cand1_T}}{\mathrm{Cul1_T}} \ll 1$$

expecting that in the limit $\rho \ll 1$ the solution of Eq. (S18) reduces to the expression in the second line of the approximate solution in Eq. (S16), i.e.

$$y_1 \approx \frac{\mathrm{SR1_T}}{\mathrm{SR1_T} + \mathrm{SR2_T}}\mathrm{Cul1_T} = \frac{\mathrm{Cul1_T}}{1+\lambda}\,, \quad \mathrm{Cand1_T} \ll \mathrm{Cul1_T}. \tag{S32}$$

Comparing this expression with the rescaled variable y in Eq. (S17) suggests to expand the solution of Eq. (S18) in the form

$$y = 1 + \rho z_1 + \mathcal{O}(\rho^2)\,. \tag{S33}$$

Substituting this expansion into Eq. (S18) and solving for z_1 leads to

$$z_1 = -\frac{K'_{sr} + \mathrm{SR_T} - \mathrm{Cul1_T}}{K'_{sr} + \left(1 + \frac{\eta K'_{sr}}{\mathrm{Cul1_T}}\right)(\mathrm{SR_T} - \mathrm{Cul1_T})}, \quad \mathrm{SR_T} > \mathrm{Cul1_T} \tag{S34}$$

Together, Eqs. (S33) and (S34) yield the expression in Eq. (S21).

S1.2.4 Derivation of Eq. (S23)

To study the behavior of the solution of Eq. (S18) when $\mathrm{Cand1_T} \gg \mathrm{Cul1_T}$ we introduce the small parameter

$$\tau = \frac{1}{\rho} = \frac{\mathrm{Cul1_T}}{\mathrm{Cand1_T}} \ll 1 \tag{S35}$$

and rewrite Eq. (S18) in the form

$$\tau^2 y^4 + 2y^3\tau\left(1+\tau\left(\frac{\eta K'_{sr}}{\mathrm{Cul1_T}}-1\right)\right)+b_2y^2+b_1y+\tau^2\left(\frac{\eta K'_{sr}}{\mathrm{Cul1_T}}\right)^2\frac{\mathrm{SR_T}}{\mathrm{Cul1_T}}=0 \tag{S36}$$

where b_2 and b_1 are given by

$$\begin{aligned} b_2 &= -\left[\tau^2\frac{\eta K'_{sr}}{\mathrm{Cul1_T}}\left(\frac{K'_{sr}+\mathrm{Cul1_T}+\mathrm{SR_T}}{\mathrm{Cul1_T}}\right)-\left(1+\tau\left(\frac{\eta K'_{sr}}{\mathrm{Cul1_T}}-1\right)\right)^2\right] \\ b_1 &= -\frac{\eta K'_{sr}}{\mathrm{Cul1_T}}\left[(1-\tau)\frac{\mathrm{Cul1_T}+\mathrm{SR_T}+K'_{sr}}{\mathrm{Cul1_T}}+\tau\frac{\eta K'_{sr}}{\mathrm{Cul1_T}}\left(1+\frac{\mathrm{SR_T}}{\mathrm{Cul1_T}}\right)\right]. \end{aligned} \tag{S37}$$

Comparing the magnitude of the different terms suggests to expand the solution in the form

$$y=\tau z_0+\mathcal{O}(\tau^2)\,. \tag{S38}$$

Substituting this expansion into Eq. (S37) leads to the quadratic equation

$$z_0^2 - z_0\frac{\eta K'_{sr}}{\mathrm{Cul1_T}}\frac{\mathrm{Cul1_T}+\mathrm{SR_T}+K'_{sr}}{\mathrm{Cul1_T}}+\left(\frac{\eta K'_{sr}}{\mathrm{Cul1_T}}\right)^2\frac{\mathrm{SR_T}}{\mathrm{Cul1_T}}=0$$

which shows that z_0 is of the form

$$z_0=\frac{\eta K'_{sr}}{\mathrm{Cul1_T}}z \tag{S39}$$

where z solves the quadratic equation

$$z^2 - z\frac{\mathrm{Cul1_T}+\mathrm{SR_T}+K'_{sr}}{\mathrm{Cul1_T}}+\frac{\mathrm{SR_T}}{\mathrm{Cul1_T}}=0.$$

In the limit $K'_{sr} \gg \min(\mathrm{Cul1_T}, \mathrm{SR_T})$ one may approximate the solution of this equation by balancing the linear with the constant term [2] which leads to

$$z \approx \frac{\mathrm{SR_T}}{\mathrm{Cul1_T}+\mathrm{SR_T}+K'_{sr}}\,. \tag{S40}$$

Combining (S38) - (S40) yields the expression in Eq. (S23).

S1.2.5 Derivation of Eq. (S24)

To study the behavior of the solution of Eq. (S18) in the limit $\eta \ll 1$ we substitute the expansion

$$y = z_0 + \eta z_1 + \mathcal{O}(\eta^2) \tag{S41}$$

into Eq. (S18). To lowest order, this yields the equation

$$\left[z_0^2 + 2z_0 \frac{\mathrm{Cand1_T} - \mathrm{Cul1_T}}{\mathrm{Cul1_T}} + \left(\frac{\mathrm{Cand1_T} - \mathrm{Cul1_T}}{\mathrm{Cul1_T}} \right)^2 \right] z_0^2 = 0,$$

which has the 2 double roots

$$z_0^{(1,2)} = 0 \tag{S42}$$

$$z_0^{(3,4)} = \frac{\mathrm{Cul1_T} - \mathrm{Cand1_T}}{\mathrm{Cul1_T}}, \quad \mathrm{Cul1_T} > \mathrm{Cand1_T}. \tag{S43}$$

Since y must be positive the solution in Eq. (S43) is only valid in the region $\mathrm{Cul1_T} > \mathrm{Cand1_T}$ where it agrees with the expression derived in Eq. (S22).

To find a solution in the region $\mathrm{Cand1_T} > \mathrm{Cul1_T}$ we consider the $\mathcal{O}(\eta)$ equation for $z_0^{1,2} = 0$ which reads

$$z_1^2 - z_1 \frac{K'_{sr}}{\mathrm{Cul1_T}} \frac{K'_{sr} + \mathrm{Cul1_T} + \mathrm{SR_T}}{\mathrm{Cand1_T} - \mathrm{Cul1_T}} + \left(\frac{K'_{sr}}{\mathrm{Cul1_T}} \right)^2 \frac{\mathrm{SR_T Cul1_T}}{(\mathrm{Cand1_T} - \mathrm{Cul1_T})^2} = 0\,. \tag{S44}$$

Hence, z_1 is of the form

$$z_1 = \frac{K'_{sr}}{\mathrm{Cul1_T}} \frac{z}{\mathrm{Cand1_T} - \mathrm{Cul1_T}}, \quad \mathrm{Cand1_T} > \mathrm{Cul1_T} \tag{S45}$$

where z solves the quadratic equation

$$z^2 - (K'_{sr} + \mathrm{Cul1_T} + \mathrm{SR_T})\, z + \mathrm{SR_T Cul1_T} = 0.$$

Similary as before, if $K'_{sr} \gg \min(\mathrm{Cul1_T}, \mathrm{SR_T})$ we may approximate z by

$$z \approx \frac{\mathrm{Cul1_T SR_T}}{K'_{sr} + \mathrm{Cul1_T} + \mathrm{SR_T}}, \quad \mathrm{Cand1_T} > \mathrm{Cul1_T}. \tag{S46}$$

Combining (S41), (S42) (S45) and (S46) yields the approximate solution in Eq. (S24).

S2 Time scale analysis

The approach to the steady state is governed by the leading eigenvalue of the Jacobian of the ODE system in Eq. (S6), i.e. by the eigenvalue with the smallest absolute magnitude. The analysis is greatly simplified in the new coordinates (Eqs. S2 - S4) because the derivatives of the right-hand sides for the equations for s_1 and s_2 vanish at steady state where $s_1 = s_2 = 0$. As a result, the Jacobian (evaluated at the steady state) assumes a lower triangular form

$$J_s = \begin{pmatrix} S & 0 \\ T & U \end{pmatrix} \tag{S47}$$

where S is a 2×2 matrix given by

$$S = \begin{pmatrix} -\frac{1}{\kappa}\left(1 + \frac{[Cul1]}{K_{sr}}\right) - \beta\frac{[Cand1]}{\eta K'_{sr}} & \beta - \frac{1}{\kappa}\frac{[Cul1]}{K_{sr}} \\ \beta\frac{[Cand1]}{\eta K'_{sr}} - \frac{\sigma-(1+\lambda)x}{K'_{sr}} & -\left(1 + \beta + \frac{\sigma-(1+\lambda)x}{K'_{sr}}\right) \end{pmatrix} k'_{sr}\,. \tag{S48}$$

The parameter $\kappa = k'_{sr}/k_{sr} \sim \mathcal{O}(10^6)$ (cf. Table 1) is called the kinetic coupling parameter [3], and its reciprocal value is naturally a small number for systems with exchange activity. The other two matrices in Eq. (S47), T and U, are of dimension 3×2 and 3×3, respectively. Due to the lower triangular form of J it is clear that the eigenvalues of J are determined by the eigenvalues of S and U. Specifically, we will show that the leading eigenvalue is contained in S, i.e. as $\text{Cand1}_\text{T}/K'_{sr} \to 0$ the matrix S has one eigenvalue of $\mathcal{O}(\kappa^{-1})$ which corresponds to the slow dissociation of Cul1.SR complexes in the absence of Cand1.

S2.1 Eigenvalues of S

To compute the eigenvalues of S we employ the approximations

$$\begin{aligned} \sigma &\approx \text{Cul1}_\text{T} \\ [Cul1] &= \text{Cul1}_\text{T} - \sigma \approx 0 \\ [Cand1] &\approx \text{Cand1}_\text{T} - \text{Cul1}_\text{T} + (1+\lambda)y_1 \end{aligned} \tag{S49}$$

which follow from Eq. (S7) together with the first line in Eq. (S13). Substituting these expressions into the characteristic equation

$$\det(S - \rho \cdot \text{I}) = 0$$

yields

$$\left(\frac{\rho}{k'_{sr}}\right)^2 + \frac{\rho}{k'_{sr}} p_\kappa + q_\kappa = 0 \tag{S50}$$

where p_κ and q_κ are given by

$$p_\kappa = 1 + \frac{\text{Cul1}_\text{T} - (1+\lambda)\,x}{K'_{sr}} + \beta\left(1 + \frac{\text{Cand1}_\text{T} - \text{Cul1}_\text{T} + (1+\lambda)\,y_1}{\eta K'_{sr}}\right) + \frac{1}{\kappa} \quad \text{(S51)}$$

$$q_\kappa = \beta\left(1 + \frac{\text{Cand1}_\text{T} - \text{Cul1}_\text{T} + (1+\lambda)\,y_1}{\eta K'_{sr}}\right)\left(1 + \frac{\text{Cul1}_\text{T} - (1+\lambda)\,x}{K'_{sr}}\right) - \beta + \frac{1}{\kappa}\left(1 + \beta + \frac{\text{Cul1}_\text{T} - (1+\lambda)\,x}{K'_{sr}}\right). \quad \text{(S52)}$$

As will be shown below the term $\sim \kappa^{-1}$ in Eq. (S51) becomes important when $\text{Cand1}_\text{T}/K'_{sr} \to 0$. However, when $\text{Cand1}_\text{T}/K'_{sr} \gg \kappa^{-1}$ the eigenvalues of S are well approximated by

$$\rho_S^\pm = -\frac{p_\infty}{2} \pm \sqrt{\frac{p_\infty^2}{4} - q_\infty} \quad \text{(S53)}$$

with p_∞ and q_∞ given by

$$p_\infty = 1 + \frac{\text{Cul1}_\text{T} - (1+\lambda)\,x}{K'_{sr}} + \beta\left(1 + \frac{\text{Cand1}_\text{T} - \text{Cul1}_\text{T} + (1+\lambda)\,y_1}{\eta K'_{sr}}\right) \quad \text{(S54)}$$

$$q_\infty = \beta\left(1 + \frac{\text{Cand1}_\text{T} - \text{Cul1}_\text{T} + (1+\lambda)\,y_1}{\eta K'_{sr}}\right)\left(1 + \frac{\text{Cul1}_\text{T} - (1+\lambda)\,x}{K'_{sr}}\right) - \beta\,. \quad \text{(S55)}$$

S2.1.1 Asymptotic analysis for $\text{Cand1}_\text{T} \gg \text{Cul1}_\text{T}$

To study the behavior of the eigenvalues defined by Eqs. (S50) - (S52) in the limit $\text{Cand1}_\text{T} \gg \text{Cul1}_\text{T}$ we recall from Eqs. (S23) and (S24) the approximate solution

$$y_1 \approx \frac{1}{1+\lambda}\frac{\text{Cul1}_\text{T}}{\text{Cand1}_\text{T}}\frac{\eta K'_{sr}\text{SR}_\text{T}}{\text{Cul1}_\text{T} + \text{SR}_\text{T} + K'_{sr}} \quad \text{(S56)}$$

which shows that

$$\lim_{\text{Cand1}_\text{T} \gg \text{Cul1}_\text{T}} y_1 \approx 0. \quad \text{(S57)}$$

In contrast, from the first line in Eq. (S14) and Eq. (S56) it follows that x approaches a finite value given by

$$\lim_{\text{Cand1}_\text{T} \gg \text{Cul1}_\text{T}} x \approx \frac{1}{1+\lambda}\frac{\text{Cul1}_\text{T}\text{SR}_\text{T}}{\text{Cul1}_\text{T} + \text{SR}_\text{T} + K'_{sr}}\,. \quad \text{(S58)}$$

Together, this shows that in the limit $\mathrm{Cand1_T} \gg \mathrm{Cul1_T}$ the terms proportional to $\mathrm{Cand1_T}$ dominate in the coefficients p_∞ (Eq. S54) and q_∞ (Eq. S55), i.e.

$$p_\infty \approx \beta \frac{\mathrm{Cand1_T}}{\eta K'_{sr}} \tag{S59}$$

$$q_\infty \approx \beta \frac{\mathrm{Cand1_T}}{\eta K'_{sr}} \left(1 + \frac{\mathrm{Cul1_T} - (1+\lambda)x}{K'_{sr}}\right) . \tag{S60}$$

Hence, for $\mathrm{Cand1_T} \gg \mathrm{Cul1_T}$ the quadratic term may be neglected against the linear and the constant term in Eq. (S50) so that the leading eigenvalue can be approximated by

$$\frac{\rho_l}{k'_{sr}} \approx -\frac{q_\infty}{p_\infty} \approx -\left(1 + \frac{\mathrm{Cul1_T} - (1+\lambda)\,x}{K'_{sr}}\right) . \tag{S61}$$

Substituting for x the approximate solution derived in Eq. (S58) yields the expression in Eq. (9) of the main text.

S2.1.2 Asymptotic analysis for $\mathbf{Cand1_T} \ll \mathbf{Cul1_T}$

To study the behavior of the eigenvalues when $\mathrm{Cand1_T} \ll \mathrm{Cul1_T}$ we employ the approximate solutions

$$\begin{aligned} y_1 &\approx \frac{1}{1+\lambda}\left(\mathrm{Cul1_T} - \mathrm{Cand1_T}\right) \\ x &\approx y_1 \end{aligned} \tag{S62}$$

which follow from the second line in Eq. (S16) in conjunction with the first line of Eq. (S14). Substituting these expressions into Eqs. (S51) and (S52) yields

$$p' = 1 + \frac{\mathrm{Cand1_T}}{K'_{sr}} + \beta + \frac{1}{\kappa} \tag{S63}$$

$$q' = \beta \frac{\mathrm{Cand1_T}}{K'_{sr}} + \frac{1}{\kappa}\left(1 + \beta + \frac{\mathrm{Cand1_T}}{K'_{sr}}\right) . \tag{S64}$$

To solve Eq. (S50) in the limit $\mathrm{Cand1_T} \ll \mathrm{Cul1_T}$ with p and q being replaced by p' and q' we set

$$\varepsilon = \frac{\mathrm{Cand1_T}}{K'_{sr}} \ll 1$$

and expand the solution as

$$v = \frac{\rho}{k'_{sr}} = v_0 + \varepsilon v_1 + \mathcal{O}(\varepsilon^2) .$$

To lowest and first order this yields the equations

$$\begin{aligned} v_0^2 + v_0 \left(1 + \beta + \frac{1}{\kappa}\right) + \frac{1}{\kappa}(1+\beta) &= 0 \\ 2v_0 v_1 + v_0 + v_1 \left(1 + \beta + \frac{1}{\kappa}\right) + \beta + \frac{1}{\kappa} &= 0. \end{aligned} \tag{S65}$$

The equation for v_0 has the solutions

$$v_0^{(\pm)} = -\frac{1+\beta+\kappa^{-1}}{2} \pm \frac{|1+\beta-\kappa^{-1}|}{2}.$$

Since $\kappa^{-1} \ll 1$ the expression inside the modulus is always positive $(1+\beta-\kappa^{-1} > 0)$ so that the two solutions for v_0 read

$$v_0^{(+)} = -\kappa^{-1} \quad \text{and} \quad v_0^{(-)} = -(1+\beta)\,. \tag{S66}$$

Substituting these expressions into Eq. (S65) yields for v_1 the two solutions

$$v_1^{(+)} = -\frac{\beta}{1+\beta-\kappa^{-1}} \quad \text{and} \quad v_1^{(-)} = -\frac{1-\kappa^{-1}}{1+\beta-\kappa^{-1}}\,. \tag{S67}$$

This shows that, neglecting terms of $\mathcal{O}(\kappa^{-1})$ in $v_1^{(\pm)}$, the eigenvalues of S can be approximated by

$$\rho_1 \approx k'_{sr} v^{(+)} \approx -k'_{sr}\left(\frac{1}{\kappa} + \frac{\beta}{1+\beta}\frac{\mathrm{Cand1_T}}{K'_{sr}}\right) \tag{S68}$$

$$\rho_2 \approx k'_{sr} v^{(-)} \approx -k'_{sr}\left(1+\beta + \frac{1}{1+\beta}\frac{\mathrm{Cand1_T}}{K'_{sr}}\right). \tag{S69}$$

Hence, $\rho_1 = \rho_l$ is the leading eigenvalue (Fig. B) whose modulus is given in Eq. (8) of the main text. Indeed, as $\mathrm{Cand1_T}/K'_{sr} \to 0$ the leading eigenvalue becomes equal to the spontaneous dissociation rate constant ($\rho_l \to -k'_{sr}/\kappa = -k_{sr}$) while $\rho_2 \to -k'_{sr}(1+\beta)$ remains of $\sim \mathcal{O}(k'_{sr})$.

S2.2 Eigenvalues of U

To compute the leading order for the eigenvalues of U we decompose the matrix U as

$$U = U_0 + \frac{U_1}{\kappa} \tag{S70}$$

with U_0 and U_1 being given by

$$U_0 = \begin{pmatrix} -\left(1 + \frac{(1+\lambda)[SR1]+\sigma-(1+\lambda)x}{K'_{sr}}\right) & 1 & \frac{[SR1]}{K'_{sr}} \\ \beta & -\beta\left(1 + \frac{[Cand1]+(1+\lambda)y_1}{\eta K'_{sr}}\right) & -\beta\frac{y_1}{\eta K'_{sr}} \\ 0 & 0 & 0 \end{pmatrix} k'_{sr} \tag{S71}$$

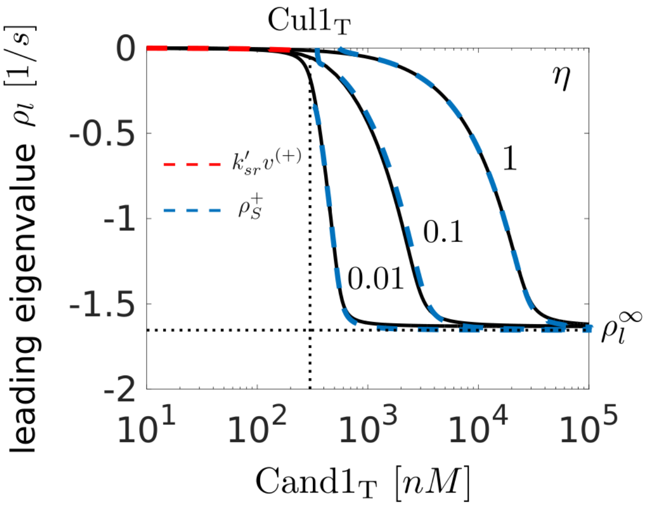

Fig B: Leading eigenvalue as a function of Cand1_T for increasing values of η. Black solid lines denote numerical computations of the leading eigenvalue. The red dotted line represents the approximation $\rho_l = k'_{sr}v^{(+)}$ (Eq. S68), which is the same for all values of η. The blue curves represent approximations of the leading eigenvalue for $\text{Cand1}_\text{T} > \text{Cul1}_\text{T}$. They were computed from ρ_S^+ in (Eq. S53) with the y in $y_1 = \text{Cul1}_\text{T} \cdot y/(1+\lambda)$ being replaced by $y_{l\eta}$ (Eq. S24). The vertical dotted line indicates the concentration of Cul1 whereas the horizontal dotted line corresponds to limiting value of the leading eigenvalue ρ_l^∞ as shown in Eq. (9) of the main text. Other parameters are listed in Table 1.

and

$$U_1 = \begin{pmatrix} -\frac{[Cul1]}{K_{sr}} & -1 & -\frac{[SR1]}{K_{sr}} \\ -\frac{[Cul1]}{K_{sr}} & -1 & -\frac{[SR1]}{K_{sr}} \\ \alpha\left(1+\lambda\right) - \frac{(1+\lambda)[Cul1]}{K_{sr}} & \alpha\frac{(1+\lambda)[Cul1]}{\eta K_{sr}} - (1+\lambda) & -\left(\alpha\left(1+\frac{[Cul1]+[Cand1]}{\eta K_{sr}}\right)+\frac{(1+\lambda)[SR1]}{K_{sr}}\right) \end{pmatrix} k'_{sr}\,. \tag{S72}$$

From this decomposition it is clear that in the limit $\kappa^{-1} \ll 1$ two of U's eigenvalues are determined by the eigenvalues of the upper 2×2 block of U_0 (cf. Eq. S71), i.e.

$$U_0^{2\times 2} = \begin{pmatrix} -\left(1+\frac{(1+\lambda)(\mathrm{SR1_T}-2x)+\mathrm{Cul1_T}}{K'_{sr}}\right) & 1 \\ \beta & -\beta\left(1+\frac{\mathrm{Cand1_T}-\mathrm{Cul1_T}+2(1+\lambda)y_1}{\eta K'_{sr}}\right) \end{pmatrix} k'_{sr} \tag{S73}$$

where we have already substituted the conservation relations (Eqs. S7) together with the relations in Eq. (S49). Using that $(1+\lambda)\mathrm{SR1_T} = \mathrm{SR_T}$ and substituting for x and y_1 their steady state values in the limit $\mathrm{Cand1_T} \ll \mathrm{Cul1_T}$ (Eq. S62) the characteristic equation can be written in the form

$$\left(\frac{\rho}{k'_{sr}}\right)^2 + \left(\frac{\rho}{k'_{sr}}\right)p + q = 0 \tag{S74}$$

with p and q given by

$$p = 1 + \frac{\mathrm{SR_T} - \mathrm{Cul1_T} + 2\mathrm{Cand1_T}}{K'_{sr}} + \beta\left(1 + \frac{\mathrm{Cul1_T} - \mathrm{Cand1_T}}{\eta K'_{sr}}\right) \tag{S75}$$

$$q = \beta\left(1 + \frac{\mathrm{Cul1_T} - \mathrm{Cand1_T}}{\eta K'_{sr}}\right)\left(1 + \frac{\mathrm{SR_T} - \mathrm{Cul1_T} + 2\mathrm{Cand1_T}}{K'_{sr}}\right) - \beta\,. \tag{S76}$$

For small $\beta \ll 1$ (cf. Table 1) the solutions of Eq. (S74) can be approximated by (see below)

$$\rho_3 \approx -k'_{sr}\beta\left(1 + \frac{\mathrm{Cul1_T} - \mathrm{Cand1_T}}{\eta K'_{sr}} - \frac{K'_{sr}}{K'_{sr} + \mathrm{SR_T} - \mathrm{Cul1_T} + 2\mathrm{Cand1_T}}\right) \tag{S77}$$

$$\rho_4 \approx -k'_{sr}\left(1 + \frac{\mathrm{SR_T} - \mathrm{Cul1_T} + 2\mathrm{Cand1_T}}{K'_{sr}}\right) - k'_{sr}\frac{\beta K'_{sr}}{K'_{sr} + \mathrm{SR_T} - \mathrm{Cul1_T} + 2\mathrm{Cand1_T}}. \tag{S78}$$

Hence, in the limit $\mathrm{Cand1_T} \ll \mathrm{Cul1_T}$ the eigenvalues remain of $\mathcal{O}(k'_{sr}\beta)$ and $\mathcal{O}(k'_{sr})$, respectively.

The 5th eigenvalue of J_s (Eq. S47) is determined by U_1 in Eq. (S72). To this end we note that, since $[Cul1] \approx 0$ (cf. Eq. S49), the components $\kappa^{-1}U_1(3,1)$ and $\kappa^{-1}U_1(3,2)$ are of $\mathcal{O}(\kappa^{-1})$ and can thus be neglected against $\kappa^{-1}U_1(3,3)$ which is actually of the same order of magnitude as the components of U_0. Under these conditions $\kappa^{-1}U_1(3,3)$ can be used as an approximation for the 5th eigenvalue. To see this more explicitly we employ the relations

$$[Cul1] \approx 0 \quad \text{and} \quad [Cand1] \approx 0 \tag{S79}$$

which follow from Eqs. (S49) and the steady state approximations for y_1 (Eq. S62). Using that $[SR1] = \mathrm{SR1_T} - x$ (cf. Eq. S7) and $x \approx y_1$ (Eq. S62) we obtain

$$\begin{aligned}\rho_5 \approx \kappa^{-1} U_1(3,3) &\approx -\kappa^{-1}\left(\alpha + \frac{(1+\lambda)\,(\mathrm{SR1_T} - x)}{K_{sr}}\right) k'_{sr} \\ &\approx -\frac{\tau}{\kappa} k'_{sr} \frac{(\mathrm{SR_T} - \mathrm{Cul1_T} + \mathrm{Cand1_T})}{K'_{sr}} \,. \end{aligned} \tag{S80}$$

Here, we have introduced the thermodynamic coupling parameter $\tau = K'_{sr}/K_{sr}$ [3] which, for the Cand1 cycle, is of the same order of magnitude as κ. Indeed, from the parameter values in Table 1 it follows that $\tau = 2\kappa$ showing that ρ_5 is of the same order of magnitude as ρ_4 in Eq. (S78).

S2.3 Derivation of Eqs. (S77) and (S78)

To derive approximate solutions of Eq. (S74) in the limit $\beta \ll 1$ we rewrite Eq. (S74) in the form

$$z^2 + z(A + \beta B) + \beta(AB - 1) = 0, \quad z = \frac{\rho}{k'_{sr}} \tag{S81}$$

with A and B being given by

$$\begin{aligned} A &= 1 + \frac{\mathrm{SR_T} - \mathrm{Cul1_T} + 2\mathrm{Cand1_T}}{K'_{sr}} \\ B &= 1 + \frac{\mathrm{Cul1_T} - \mathrm{Cand1_T}}{\eta K'_{sr}} \,. \end{aligned}$$

To find an approximate solution of the quadratic equation Eq. (S81) we substitute the expansion

$$z = z_0 + \beta z_1 + \mathcal{O}(\beta^2) \tag{S82}$$

into Eq. (S81) which yields the set of equations

$$z_0\,(z_0 + A) = 0 \tag{S83}$$

$$2z_0 z_1 + z_0 B + z_1 A + AB - 1 = 0 \,. \tag{S84}$$

The solutions of Eq. (S83) are given by

$$z_0^{(1)} = 0 \quad \text{and} \quad z_0^{(2)} = -A \,. \tag{S85}$$

Substituting these expressions into Eq. (S84) and solving for z_1 yields

$$z_1^{(1)} = -\left(B - \frac{1}{A}\right) \quad \text{and} \quad z_1^{(2)} = -\frac{1}{A} \,, \tag{S86}$$

so that

$$\rho_3 = k'_{sr} z^{(1)} \approx -k'_{sr}\beta \left(B - \frac{1}{A} \right) \quad \text{and} \quad \rho_4 = k'_{sr} z^{(2)} \approx -k'_{sr} \left(A + \frac{\beta}{A} \right) \tag{S87}$$

which agrees with Eqs. (S77) and (S78).

References

[1] E. J. Bennett, J. Rush, S. P. Gygi, J. W. Harper, Dynamics of cullin-RING ubiquitin ligase network revealed by systematic quantitative proteomics. *Cell* **143**, 951-965 (2010).

[2] R. Straube, Reciprocal regulation as a source of ultrasensitivity in two-component systems with a bifunctional sensor kinase. *PLoS Comput. Biol.* **10**, e10036147 (2014).

[3] R. S. Goody, W. Hofmann-Goody, Exchange factors, effectors, GAPs and motor proteins: common thermodynamic and kinetic principles for different functions. *Eur. Biophys. J.* **31**, 268-274 (2002).
